# The Role and Function of Regulatory T Cells in *Toxoplasma gondii*-Induced Adverse Pregnancy Outcomes

**DOI:** 10.1155/2021/8782672

**Published:** 2021-08-18

**Authors:** Xuyang Gao, Yue Zhong, Yifan Liu, Runmin Ding, Jinling Chen

**Affiliations:** Department of Pathogen Biology, School of Medicine, Nantong University, Nantong, 226001 Jiangsu, China

## Abstract

Infection with *Toxoplasma gondii* (*T. gondii*) during the pregnant period and its potentially miserable outcomes for the fetus, newborn, and even adult offspring continuously occur worldwide. People acquire infection through the consumption of infected and undercooked meat or contaminated food or water. *T. gondii* infection in pregnant women primarily during the gestation causes microcephaly, mental and psychomotor retardation, or death. Abnormal pregnancy outcomes are mainly associated with regulatory T cell (Treg) dysfunction. Tregs, a special subpopulation of T cells, function as a vital regulator in maintaining immune homeostasis. Tregs exert a critical effect on forming and maintaining maternal-fetal tolerance and promoting fetal development during the pregnancy period. Forkhead box P3 (Foxp3), a significant functional factor of Tregs, determines the status of Tregs. In this review, we summarize the effects of *T. gondii* infection on host Tregs and its critical transcriptional factor, Foxp3.

## 1. Introduction

**Figure 1:**
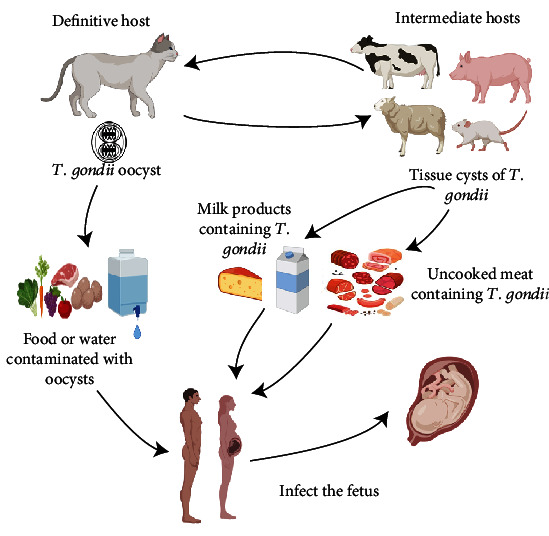
Life cycle and spread of *T. gondii* between people and animal. Feline is the definitive host. Intermediate hosts will infect by ingesting the water source of *T. gondii* oocyst deposited in cat feces or animal meat and milk products containing cysts or pseudocysts. Pregnant women infected during the gestation period will bring about adverse outcomes because *T. gondii* can be transmitted to the fetus through the placenta.

*T. gondii* is an obligate intracellular parasite with a complicated life cycle, belonging to apicomplexa. *T. gondii* requires two hosts, mammals including human acting as intermediate hosts and cats, which are definitive hosts [1]. People acquire infection by eating undercooked meats or dairy products which contain cysts or pseudocyst of *T. gondii* or by contacting with water contaminated with feces of cats that involve *T. gondii* oocysts [2]. *T. gondii* in an individual with normal immunity is in a state of latent infection and produces no obvious clinical effect. Nevertheless, an individual with compromised immunity possibly suffers from ocular toxoplasmosis and fatal diseases of the central nervous system like encephalitis. Contracting *T. gondii* during the pregnancy, which is a state of immunological tolerance, might be a lethal factor for the fetus. The overall risk of congenital infection from primary *T. gondii* infection varies from 20% to 50% without treatment [3]. Based on the seroprevalence study in Central and Southern Italy from 2013 to 2017, the prevalence of pregnant women remains 13.8%, although pregnant women are conscious of the importance of hygiene and diet to prevent primary *T. gondii* infection [4]. *T. gondii* tachyzoites infect fetuses and cause potentially tragic outcomes such as microcephaly, intrauterine growth restriction, or death [5] (Figure 1). And the severity of *T. gondii* infection is closely associated with gestational age [6]. Chorioallantoic attachment did not occur until embryonic day (E) 8.5 during the development of mouse placenta. At this stage, trophoblast cells of the chorionic plate and mesoderm cells of allantois begin to interdigitate to generate villi [7]. Villous explantation has high resistance to pathogen infection [8]. Therefore, *Toxoplasma* infection, which occurs in the early pregnancy, enhances the possibility of miscarriage.

Normal pregnancy is a special immune phenomenon, similar to allotransplantation. Many mechanisms protect the fetus from the maternal immune system, including the nonclassical MHC molecules expressed on trophoblast cells, the complement system, tryptophan catabolism by the action of enzyme indoleamine 2,3-dioxygenase (IDO), T cell apoptosis, and suppressive function of CD4^+^ CD25^+^ Tregs [9]. Among them, Tregs are documented as important regulators in maintaining normal pregnancy [10]. Tregs modulate the immune response mainly by secreting inhibitory factors such as transforming growth factor-*β* (TGF-*β*) and interleukin-10 (IL-10) or inhibiting inflammatory cytokines produced by Th1/Th17 cells, such as interferon-*γ* (IFN-*γ*), IL-17, and IL-23, in order to protect against their harmful effects [11, 12].

Pregnant women infected with *T. gondii* during the gestation period will lead to decidual Treg depletion in number and downregulation in function on the maternal-fetal interface [13]. In our laboratory, previous research has shown that the decrease in the number and function of Tregs of pregnant mice results from *T. gondii* excreted-secreted antigens [14], which break immune tolerance of normal pregnancy and finally cause abortion during early pregnancy [15]. In this article, we review the role of Tregs and the underlying mechanism in *T. gondii*-induced adverse pregnancy outcomes.

## 2. Destructions of Placental Structure by *T. gondii* Infection

**Figure 2:**
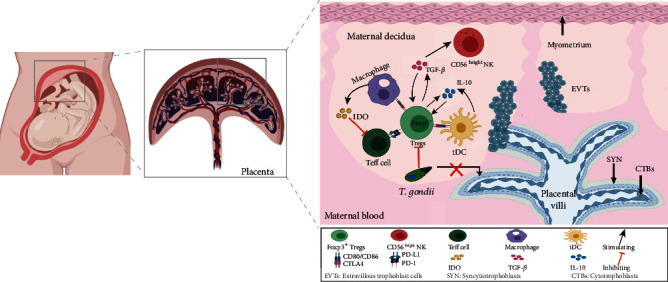
Mechanism of maternal-fetal immune regulation. The maternal-fetal interface is composed of CTBs and SYN, formed by the fusion of underlying CTBs. SYN, the key interface between the blood and fetal barrier, is highly resistant to *T. gondii*. *T. gondii* rarely goes across SYN. *T. gondii* infection affects maternal-fetal immune regulation by affecting maternal regulatory immune cells, mainly by inhibiting Tregs. CTBs: cytotrophoblasts; SYN, syncytiotrophoblasts; EVTs: extravillous trophoblasts; Teff cell: effector T; tDC: tolerance-inducing DC; images were created with BioRender.

Human placenta, a critical organ with multiple functions like endocrine and immune reaction, consists of its umbilical cord, amnion, parenchyma, and chorion. Chorion differentiates into floating and anchoring villi. Floating villi are formed by an inner layer of cytotrophoblasts (CTBs) where a layer of syncytiotrophoblasts (SYN) covers, while anchoring villi attach itself to maternal decidual tissue via extravillous trophoblasts (EVTs). EVTs straightly invade the decidua basalis and thus anchor the placenta into the uterine implantation site, in which the EVTs directly contact with maternal immune cells. The maternal-fetal interface is composed of CTBs and SYN that are formed via the fusion of the underlying CTBs. SYN on both floating and anchoring villi constitutes the outermost cell layer and thereby forms the critical interface between maternal and fetal blood [16]. The syncytiotrophoblast layer has high resistance to *T. gondii* infection. *T. gondii* rarely goes across the syncytiotrophoblast layer *in vivo* [17]. When syncytium is damaged, it would allow for pathogen to enter the villous core [18]. The influence might be dependent on the gestation time as well, for the layer of subsyncytial CTBs becomes thinner and discontinuous in part after the first trimester. Although *T. gondii* replicates well in underlying subsyncytial CTBs, it fails to colonize SYN [18]. Those indicate that *T. gondii* might invade subsyncytial CTBs only if the syncytiotrophoblast layer ruptures **(**Figure 2**)**.

In the process of placentation, trophoblast cells from implanted blastocyst invade the mother's endometrium. Endometrial stromal cells differentiate through a process called decidualization, which contributes to trophoblast invasion [19]. According to the contact pattern between the trophoblast and endometrium, the placentae of eutherians are classified in epitheliochorial, endotheliochorial, and hemochorial placentae. In hematochorionic placentas of human and mice, the fetal membrane is in direct contact with maternal tissue and blood [20]. To maintain successful pregnancy, the deep placentation implies proper recognition and tolerance of semiallogeneic fetuses, in which maternal immune cells play a key role. Tregs infiltrate into the decidua of pregnancy and play a crucial role in fetal tolerance, trophoblast invasion, and tissue and vascular remodeling, along with other leukocytes (macrophages, NK cells, and dendritic cells) [21].

Brito et al. infected BALB/c mice with *T. gondii* type II strain (ME49) [22]. Histopathological analysis showed that *T. gondii* was generally detected in the muscularis at the early gestation period, and a small number of *T. gondii* were found in the decidua on the 14th day of gestation. On the 18th day of gestation, necrosis appeared at the maternal-fetal interface and *T. gondii* could be observed in the placenta. 2000 freshly sporulated oocysts of *T. gondii* M4 were administered orally to Churra sheep during the pregnancy [23]. From 7 to 11 days after infection, abortion occurred in pregnant ewes. The placenta with different degrees of autolytic edema could be seen under the microscope. Histological examination revealed infarction and thrombus formation of the villi of the placental corpuscle wall, which caused fetal hypoxia damage and was related to acute abortion. Fadaam et al. found that *T. gondii* could be detected in the fetal brain, lung, and placenta. And inflammatory pathological changes of trophoblastic cells in the placenta, trophoblast edema, hemorrhage, and fibrinoid necrosis were observed, indicating that *T. gondii* during the pregnancy was transmitted to the fetus through the placenta, affecting pathological changes of placental structure and further damaging trophoblast cells of the placenta [24]. In addition, pathologic examination revealed necrotic granuloma in the villous stroma leading to fetal autolysis in pregnant women infected with *T. gondii* [25]. A normal mouse placenta consists of the maternal decidua and the fetal embryo-derived compartments, containing the junctional zone and labyrinth zone [26]. Nearly all of the embryos and placentas in pregnant mice exhibited a necrotic and hemorrhagic appearance at the early stage of pregnancy following the administration of antigens from *T. gondii* [14]. The function of the labyrinth zone in the mouse placenta is to be equivalent to that of the chorionic villus of the human placenta. The labyrinth zone of the mouse placenta displayed the classical interhemal barrier, breaking fetal blood vessels and maternal lacunae upon the administration of antigens from *T. gondii* [14]. Hence, destructions of the placental structure may partially account for the adverse pregnancy triggered by *T. gondii*.

## 3. Effects of *T. gondii* Infection on Maternal-Fetal Immune Regulation

### 3.1. Maternal-Fetal Immune Regulation

Normal pregnancy is, to a great extent, dependent on maternal immune tolerance, as the fetus consists of the tissue-specific as well as paternally inherited antigens. Balance between inactivation of alloreactive effector cells and/or clone deletion and immune suppression triggered by regulatory immune cells constitute maternal immune tolerance. Innate regulatory immune cells including alternatively activated/regenerative-type macrophages (M2), tolerance-inducing DCs (tDCs), and CD56^bright^ CD16^−^ decidual NK cells (dNK) interact with adaptive cells comprising Tregs to constitute a key network that maintain a successful pregnancy [27].

Macrophages have a capacity for immunosuppressive activity and production of cytokine besides antigen presentation. According to the function and repertoire of cytokine production, macrophages are generally classified into two significant subpopulations: M1 and M2. M1 macrophages are an inflammatory-type presenting antigen, producing proinflammatory cytokine and nitric oxide (NO) as well as reactive oxygen species (ROS). M2 macrophages, which are induced by Th2 cytokines like IL-4 and IL-13, are alternatively activated/regenerative type that exert an immunosuppressive function and promote immune tolerance and tissue remodeling at the maternal-fetal interface [21]. M2 macrophages play immunosuppressive roles by abundant production of IL-10 and IDO, accompanied with prostaglandin-E2 (PGE2) which limits the activation of cytotoxic leukocytes [28]. IDO produced by M2 is mediated by Tregs via cytotoxic T-lymphocyte-associated protein 4 (CTLA-4) expressed on Treg surface. The shift from M2 to M1 phenotype during pregnancy is linked to adverse pregnant outcomes like miscarriages or preeclampsia [29].

The DCs orchestrate T cell activation and differentiation via presenting antigen and providing costimulatory signaling. Placental formation during the early pregnancy is correlated with immature DCs with tolerogenic capacity. DCs induce Treg differentiation along with abundant production of IL-10 during the pregnancy [30]. Additionally, Tregs produce heme oxygenase-1 (HO-1) to maintain the immature state of DCs, which further induces Treg formation via higher level of IL-10 [31]. DCs produce IDO and TGF-*β* to interact with CTLA-4 expressed on Tregs, which inhibit allogen-specific T cell activity, improve Treg differentiation, and further break Treg/Teff balance.

Both uterine and decidual NK cells exert their immune-regulatory functions in the process of placental vascularization and formation during the early pregnancy. Imbalance between regulatory CD56^bright^ NK cells and cytotoxic CD56^dim^ may impair maternal immune tolerance. The decreased CD56^bright^/CD56^dim^ NK cell ratio is bound up with adverse pregnancy outcomes like recurrent pregnancy loss [32]. Tregs are implicated in the regulation of cell phenotype and generation of dNK cells via inhibiting cytotoxicity of NK cells in a TGF-*β*-dependent manner and suppressing the release of IL-15 from DCs. Similarly, TGF-*β* produced by Tregs shifts NK cell from the peripheral CD56^dim^ to decidual-like CD56^bright^ phenotype. NK cells improve Treg homeostasis via alleviating Th17 cell responses through secreting IFN-*γ* and promoting Treg development.

Early studies have shown that Th1/Th2 intercellular immune balance and Th2 cell predomination are involved in the mechanisms of maintaining normal pregnancy [33, 34]. Cytokines, secreted by Th2 like IL-4 and IL-6, can induce trophoblast cells to release hCG and stimulate the production of progesterone [35], which in turn stimulates Th2 cells to reduce the secretion of Th1 cytokines [36]. However, in knockout mouse models that cannot secrete Th2 cytokines, abortion is not always possible [37], indicating that Th2 cytokines are not essential for the maintenance of normal pregnancy [38]. In recent years, studies have suggested that Th17/Treg cell balance is closely related to the formation and maintenance of maternal-fetal tolerance [39]. Th17 mainly mediates the immune response by secreting proinflammatory cytokines like IL-17 and IL-22 and specifically expresses the transcription factors orphan nuclear receptor (ROR*γ*t) and signal transducer and activator of transcription 3 (STAT3). IL-35, a newly discovered anti-inflammatory cytokine secreted by Tregs, functions as a regulator by promoting Treg amplification and inhibiting Th17 differentiation [40]. IL-35 suppresses the production of IL-17, but the levels of IL-35 and IL-35/IL-17 in patients with recurrent abortion are significantly lower than normal [41]. It follows that the deviation of Th17 will enhance the maternal immune response to the fetus, which is not conducive to the maintenance of normal pregnancy [10].

The pathogenic effects of *T. gondii* mainly contain the direct action of *T. gondii* and the immunopathological response triggered by *T. gondii* antigen. Abortion caused by *T. gondii* infection is predominately related to the disruption of the maternal-fetal interface immune balance induced by *T. gondii* antigen in early pregnancy [42]. *T. gondii* ESA are dissoluble antigens that stick to and invade host cells in the early stage of *T. gondii* infection, and are excreted or secreted during intracellular proliferation [43]. It has strong immunogenicity [44], which can induce the host to provoke humoral and cellular immune responses and cause immune response [45]. The influence of ESA on the host is similar to the host directly infected with *T. gondii*. Pregnant mice injected with ESA could result in abortion during the early stage of pregnancy, accompanied with decreased levels of CD4^+^CD25^+^ Tregs and Foxp3 in the spleen and placenta [13]. Therefore, fetal resorption mediated by *T. gondii* is largely owing to immunopathological reaction rather than the direct effect of *T. gondii* proliferation in the uterus.

### 3.2. Characteristics and Mechanisms of Regulatory T Cells

Tregs, accounting for 5-10% of the total CD4 ^+^ T cell pool and expressing T cell receptors (TCR), are mostly distinct from that of conventional CD4^+^CD25^+^ T cells. Tregs derive from two different populations that exert synergy effect to enhance peripheral immune tolerance [46, 47]: (1) CD4^+^ CD25^+^ Foxp3^+^ natural regulatory T (nTreg) cells, enriched with an anti-self-biased TCR repertoire, differentiate from immature precursors in the thymus and enhance immune tolerance to self-antigens [48] and (2) induced regulatory T (iTreg) cells, developed from naive conventional CD4^+^CD25^+^ T cells after antigen, encounter with specific factors such as TGF-*β* and IL-2 and act as effective Tregs to suppress the immune response [49].

Tregs can be activated by self-antigens as well as non-self-antigens [50]. Activated Tregs have the capacity of inhibiting T cell proliferation in specific and nonspecific antigen manners. Notably, the inhibitory function of Tregs is not limited to the adaptive immune system but impacts the activation and function of innate immune cells such as monocytes, neutrophils, macrophages, and dendritic cells [51]. Various mechanisms by which Tregs maintain self-tolerance as well as suppress autoimmune responses and chronic inflammation are involved: (1) Tregs kill target cells via a granzyme B-dependent, perforin-independent pathway [52]; (2) Tregs modulate target cells via binding to the corresponding receptor of target cells such as CTLA-4 and PD-1 [53, 54]; (3) Tregs play immunosuppressive roles via secreting immune regulatory factors like TGF-*β*, IL-10, or IL-4 [55, 56]; and (4) Tregs inhibit target cells by exosome-carried microRNAs [57].

### 3.3. Regulatory T Cells during Normal Pregnancy

Tregs usually proliferate in the early stage of pregnancy with the enhanced immunosuppressive ability, which will continue until the end of pregnancy [58]. Aluvihare et al. firstly demonstrated an increase in the number of Tregs during normal pregnancy in an animal model, and the lack of Tregs eventually causes abortion [59]. The decreased number of Tregs was observed in mice prone to abortion, which can be prevented through adoptive transfer of Tregs from the spleen of normal pregnant mice [60]. The number of Tregs was reduced in patients prone to recurrent spontaneous abortion as well [61, 62], indicating that Tregs push forward an immense influence on maintaining normal human pregnancy. In the early pregnancy, the number of Tregs increases gradually and reaches the highest level when trophoblast cells invade the decidua, suggesting that Tregs are involved in regulating the uterine immune response to the placenta [54, 63]. Studies have shown that Tregs mainly rely on three mechanisms to promote implantation and embryo development [64]. Firstly, Tregs can prevent effector T (Teff) cells from damaging the fetus in an antigen-dependent trophoblastic cytotoxic manner by secreting IL-10, TGF-*β*, CTLA-4, and PD-1 [35, 64, 65]. Secondly, Tregs can regulate other cells like M2-type macrophages and tDCs [66]. Tregs induce M2 macrophages and tDCs to express IDO, which can decrease Th1 cells [67]. Thirdly, Tregs have vascular regulation function [68], which is crucial for normal placental development and placental pathway with sufficient maternal blood. When Tregs were deficient, changes in uterine spiral arteries and placental hemodynamics were not conducive to fetal development [67, 69]. In addition, unexplained infertility and abortion are linked to the deficiency of the number and function of Tregs [54]. The expression of Foxp3 mRNA in the endometrium is very low in patients with unexplained infertility, suggesting that the differentiation ability of uterine T cells into Treg phenotypes is impaired, thereby affecting fertility [70].

Hence, the number and function of Tregs increase during the normal pregnancy and impair in the pregnancy failure, indicating that Tregs is extremely crucial during pregnancy (Figure 2).

### 3.4. The Role of Tregs on *T. gondii* Infection-Induced Abortion

Tregs are associated with adverse pregnancy induced by *T. gondii* as well. *T. gondii* infection results in a decreased number of decidua Tregs, accompanied with decreased levels of immune-related functional molecules like IL-10 and TGF-*β* [71]. In addition, study has shown that acute *T. gondii* infection can directly inhibit Treg proliferation [72].

#### 3.4.1. *T. gondii* Induces a Decrease in the Number of Tregs

It has been found that the number of Tregs in the spleen and placenta was reduced in a *T. gondii*-infected pregnant mouse model [10]. The decreased number of Tregs is associated with apoptosis triggered by *T. gondii* infection [73]. IL-10 is an important cytokine to maintain normal pregnancy, and the hyposecretion of IL-10 in the decidua is correlated with adverse pregnancy [74]. Some studies indicate that IL-10 can regulate the expression of various apoptotic factors to prevent apoptosis [75, 76]. Lao et al. established an *T. gondii* infection animal model using recombinant IL-10 (rIL-10) and IL-10-deficient mice [77]. It was found that cleaved caspase-3 and caspase-8 were upregulated in decidual Tregs in the IL-10^−/−^ group, while those were decreased in the rIL-10 treatment group along with improved pregnant outcomes, indicating that IL-10 has the capacity of inhibiting the apoptosis of decidual Tregs and improving adverse pregnant outcomes.

The severity of adverse pregnant outcomes upon primary infection with *T. gondii* is bound up with the gestational time. *T. gondii* infection in the early stage of pregnancy can more possibly cause abortion than that in the late pregnancy in the mouse model, and the main reason is the apoptosis rate of Tregs induced by *T. gondii* infection in the early stage of pregnancy [78]. *T. gondii* infection can result in a decrease in the number of Tregs in the mouse placenta and spleen [10]. A significant decrease in mortality was observed through adoptive transfer of normal mouse CD4^+^ Tregs to *T. gondii-*infected mice [79], indicating that maintaining a certain number of Tregs is crucial to improve the adverse results caused by *T. gondii* infection.

Estradiol is implicated in several aspects of pregnancy, suggesting its indispensable role in pregnancy. Qiu et al. demonstrated that the decreased number of Tregs induced by *T. gondii* infection is attributed to Treg apoptosis mediated by *T. gondii* [78]. Compared with late pregnancy, the rate of Treg apoptosis was enhanced in the early pregnancy, accompanied with reduced PD-1 expression. Estradiol (E2) *in vitro* could provide protection against apoptosis and enhance PD-1 expression on Tregs through estradiol receptor (ER) in a dose-dependent manner. Simultaneously, E2 administration in nonpregnant mice could ameliorate the apoptosis rate of Tregs induced by *T. gondii* infection, accompanied with the potentiated expression of PD-1 on Tregs. E2 might help support the immune tolerance and improve the adverse pregnancy via targeting on Tregs. Those findings verify the role of Tregs in *T. gondii*-induced adverse pregnancy.

#### 3.4.2. *T. gondii* Induces Dysfunction of Tregs

Tregs play an immunosuppressive role through CTLA-4 and PD-1 binding to the target cell surface [80, 81] as well as secreting cytokines IL-10 and TGF-*β* [77, 82], which are important for protective tolerance induced by Tregs during the pregnancy. CTLA-4 expression in a decidual membrane is positively correlated with the secretion of anti-inflammatory cytokines, indicating the significant immunosuppressive activity of CTLA-4 at the maternal-fetal interface [83]. Additionally, the combination of CTLA-4 and its ligand CD80/CD86 can induce IDO expression, and IDO will further promote maternal-fetal immune tolerance [84]. When CTLA-4 is deficient, the function of Tregs will decrease [80]. PD-1 is another important factor for Tregs to induce fetal protection in a mouse model [85]. PD-1 binds to PD-L1 expressed on trophoblastic cells [86], which can transmit inhibitory signals down to exert immunosuppressive effects. Though PD-1 blockade has no significant effect on Treg number, it could induce the impairment of Treg function in recurrent early abortion. Blocking PD-1 by injection of monoclonal antibody can cause fetal loss in pregnant mice, which is linked with insufficiency of Treg function and amplification of Teff [87]. Research has shown that the expression levels of CTLA-4, PD-1, TGF-*β*, and IL-10 in Tregs from pregnant mice with abortion induced by *T. gondii* infection are downregulated, while the levels of the inflammatory cytokines are increased [73]. High level of IFN-*γ* instead leads to maternal immune response of fetal abortion [88], and the adoptive transfer of Tregs from healthy pregnancy mice can improve the adverse pregnant outcomes caused by *T. gondii* infection.

### 3.5. Signaling Pathways of Suppressing Foxp3 Caused by Excreted-Secreted Antigens

The continuous stability and high expression of Foxp3 are the key to the development of Tregs. Foxp3, an acknowledged character of Tregs, is implicated in the establishment and maintenance of Tregs and takes charge of maintaining immune homeostasis [89]. In patients with recurrent spontaneous abortion, the expression of Foxp3 protein in peripheral blood and decidual tissues is significantly less than that in normal pregnant women [90]. In addition, the expression of Foxp3 in women with unexplained infertility was associated with a lower number or percentage of Tregs in endometrial tissue [70]. Previous studies in our laboratory have shown that ESA could suppress Foxp3 expression both *in vivo* and *in vitro* and inhibit the function of Tregs, thereby causing abortion [14]. We all know that the regulation of Foxp3 is relatively complicated, including the TGF-*β*/Smad pathway, the interleukin-2 receptor/signal transducer and activator of transcription (IL-2R/STAT) pathway, and the phosphatidylinositol 3-kinase/protein kinase B/mammalian target of rapamycin (PI3K-AKT-mTOR) pathway.

TGF-*β* signaling plays an indispensable role in the early development of Tregs [91] and is a necessity to maintain the number of Tregs in peripheral lymphatic tissue [92]. TGF-*β*, binding with TGF-*β* type II receptor (T*β*RII), induces phosphorylation of T*β*RII and activates its kinase activity, which further activates Smad2 and Smad3 protein by phosphorylation. And then, phosphorylated Smad2 and Smad3 bind to Smad4, form the Smad complexes, and transfer into the nucleus, thereby regulating Foxp3 expression [93]. Our previous study revealed that Chinese 1 strain of *T. gondii* ESA could suppress Foxp3 by inhibiting Smad2 and Smad3 phosphorylation in pregnant mice [14]. Meanwhile, the overexpression of Smad2/Smad3/Smad4 can partially offset the inhibition of Foxp3 induced by ESA. It can be seen that ESA directly inhibits the expression of T*β*RII, suppresses the activation of Smad2/Smad3/Smad4 signaling pathway, and negatively modulates Foxp3, causing abortion. Treatment with TGF-*β* can prominently improve adverse pregnant outcomes caused by *T. gondii* infection [94]. The TGF-*β*/Smad signaling pathway can enhance the differentiation, development and function of Tregs, regulate Foxp3, and inhibit high levels of maternal-fetal inflammation triggered by *T. gondii* infection.

Besides the TGF-*β*/Smad signaling pathway, IL-2R is also essential for the development of Tregs and the transcription of Foxp3 [95]. IL-2R/Janus kinase 3 (JAK3)/STAT signaling pathway is associated with the development and functional maintenance of Tregs [96]. Binding to the corresponding receptor, heterodimerization of the cytoplasmic domain, IL-2 induces the activation of JAK3, which activates STATs by phosphorylation, mainly STAT5. A previous study has shown that ESA of *T. gondii* suppresses Foxp3 by directly inhibiting IL-2R, JAK3, and the phosphorylation of STAT3 and STAT5, while overexpression of STAT3/STAT5 can partially attenuate the inhibitory effect of ESA on Foxp3 [97]. Therefore, ESA of *T. gondii* inhibits Foxp3 via the IL-2R/JAK3/STAT signaling pathway, thus suppressing Treg function.

The PI3K-AKT-mTOR signaling pathway mediates cell proliferation, differentiation, and apoptosis [98]. Tregs are sensitive to PI3K activation, and PI3K activation will downregulate the expression of Foxp3, thus negatively affecting Treg function, while inducible T cell costimulator (ICOS) can activate negative regulators of PI3K such as TANK binding kinase 1 (TBK1) [55] to maintain the normal function of Tregs [99]. The activation of PI3K produces the second messenger phosphoinositide 3 kinase (PIP3), which binds to the intracellular signal protein AKT. Activated AKT induces the phosphorylation of mTOR, affects the expression of cytokine in T cells, and exerts a critical immunosuppression function. The PI3K-AKT-mTOR pathway negatively regulates Foxp3 via inactivating the transcription factor Forkhead O3a [100]. ESA can inhibit Foxp3 by upregulating PI3K, AKT, and mTOR [101], leading to downregulation of the immune function of Tregs.

**Figure 3:**
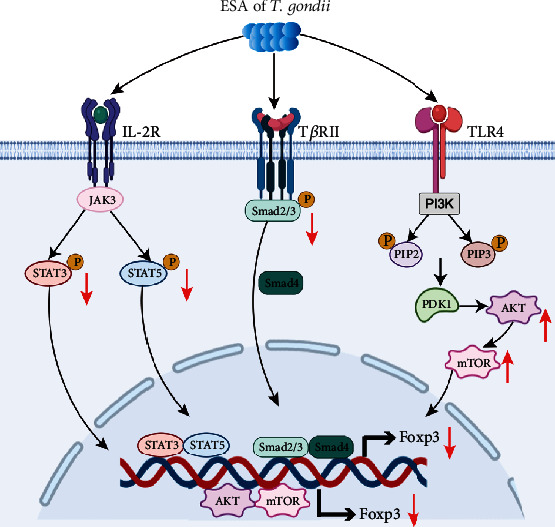
Mechanism of inhibiting Foxp3 by excreted-secreted antigens of *T. gondii*. ESA can inhibit the expression of Foxp3 by inducing the deactivation of IL-2R, inhibiting the expression of JAK3, and reducing the phosphorylation levels of STAT3 and STAT5. Furthermore, ESA may inactivate T*β*RII and further suppress the levels of p-Smad2, p-Smad3, and Smad4 in order to downregulate the expression of Foxp3. In addition, ESA can upregulate the expression of PI3K/AKT/mTOR via TLR4, resulting in the decrease of Foxp3 expression. ESA: excreted-secreted antigens; Foxp3: forkhead box p3; IL-2R: interleukin 2 receptor; JAK3: Janus kinase 3; STAT3: signal transducer and activator of transcription 3; T*β*RII: TGF-*β* type II receptor; TLR4: Toll-like receptor 4; PI3K: phosphatidylinositol 3-kinase; AKT: protein kinase B; mTOR: mammalian target of rapamycin.

Foxp3 functions as a key regulator in the development and function of Tregs. ESA of *T. gondii* can inhibit Foxp3 via suppressing the expression of T*β*RII and IL-2R, cutting the phosphorylation levels of Smads and STATs. Moreover, ESA can suppress Foxp3 by upregulating PI3K, AKT, and mTOR as well **(**Figure 3**)**. The suppression of Foxp3 expression indicates the downregulation of Treg function, leading to adverse pregnancy.

## 4. Role of Tregs in Long-Term Effects of *T. gondii* Infection on the Fetus

*T. gondii* infection largely causes abortion in the early pregnancy, whereas its infection that occurred in the late pregnancy mainly induces neuropsychiatric diseases and behavior alterations in humans and rodents [102]. *T. gondii* infection increases vulnerability to schizophrenia, which is evidenced by the fact that the risk of schizophrenia among individuals prenatally exposed to *T. gondii* was more than twice that of healthy subjects. Consistent with these results, immunoglobulin G levels of *T. gondii* were closely linked to schizophrenia risk [72]. Several underlying mechanisms are involved, including enhanced testosterone [103], increased dopamine and decreased serotonin [104], and different immune alterations [105]. Hellmer and Nystrom reported that dysregulation of infant acetylcholine, dopamine, and melatonin may be responsible for autism spectrum disorders (ASD) [106]. Immune imbalance is a causal factor of schizophrenia as well. Alterations of circulating CD4^+^ T lymphocytes were observed in individuals with schizophrenia [105]. A similar finding was demonstrated in an independent sample in which the neuroinflammation triggered by CD4^+^ T cells could impact the central nervous system [107]. Neurotransmitters like dopamine are postulated to critical regulators of T cell functions [108]. In parallel, gene variants of dopamine receptor were largely linked to the amount of CD4^+^ T cells rather than CD8^+^ T cells [109].

Tregs are susceptible to dopamine and cyclic AMP levels in lymph cells [79]. Dopamine receptor D5 (DRD5) signaling strengthens suppressive capacity of Tregs, thereby mitigating the manifestation of experimental autoimmune encephalomyelitis (EAE). Additionally, the anti-inflammatory effect of DRD5 signaling in Tregs is bound up with increased glucocorticoid-induced tumor necrosis factor receptor-related protein (GITR) expression, which can contribute to Treg expansion [110]. Simultaneously, Tregs have a neuroprotective capacity via promoting neurotrophic factor expression and repressing the synthesis of proinflammatory cytokines as well as ROS, which could impair the higher-order brain functions and thereby contribute to the progressive brain alterations [111]. Xu et al. established an animal model of maternal immune activation by the injection of *T. gondii* soluble tachyzoite antigen (STAg) on E 14.5 [112]. Consistent with our previous study, *T. gondii* antigen failed to induce abortion in the late pregnancy period [14]. At 3 days after injection, the decreased Tregs but increased Th1 and Th17 cells in the spleen of pregnant mice were observed, indicating that STAg could exert a proinflammatory T cell immune profile [112]. Offspring exposure to STAg-triggered MIA exhibited impaired-communicative capacity and anxiety-like behaviors as well as deficits in social behaviors. Isolated CD4^+^CD25^+^ Tregs from PBS-treated (_C_Tregs) and STAg-triggered MIA (_MIA_Tregs) of mother mice were intravenously transferred into adult progeny at the age of 8 weeks, respectively. Treg transfer could effectively reverse autism-related manifestations. Noteworthily, _MIA_Tregs appeared to have greater efficacy on immune suppression than _C_Tregs in the brain of offspring. *T. gondii*-activated maternal Tregs could rescue behavior abnormalities in the offspring of adult mice induced by maternal immune activation. Therefore, sufficient Tregs not only prevent against the miscarriage but improve behavior abnormalities in the offspring of adult mice induced by *T. gondii*.

## 5. Conclusions and Future Directions

*T. gondii* infection can invade the placental tissue in different ways and destroy maternal-fetal immune tolerance during the pregnancy, which can lead to maternal immune rejection, affect fetal growth, and cause abortion or other pregnancy complications. Tregs play a vital role in the immune regulation of pregnancy [113], and the decline in the number or function of Tregs is associated with adverse pregnancy. As a critical functional molecule of Tregs, Foxp3 expression directly determines the state of Tregs. Extensive studies have been done to unravel the role of Tregs in different types of adverse pregnancy through mouse models. Treg transfer might be a potential therapeutic to treat adverse pregnancy, especially behavior abnormalities in the offspring of adult mice induced by maternal immune activation. The signaling pathways regulating Foxp3 expression can be targeted to recovery from adverse pregnancy as well.

## References

[1] Lourido S. (2019). _Toxoplasma gondii_. *Trends in Parasitology*.

[2] Barragan A., David Sibley L. (2003). Migration of _Toxoplasma gondii_ across biological barriers. *Trends in Microbiology*.

[3] Ferrucci M., Dall'Ara G. (1980). Immunological indexes of receptivity and seroconversion for rubella and toxoplasmosis in the province of Ferrara, Italy. *Annali Sclavo; rivista di microbiologia e di immunologia*.

[4] Fanigliulo D., Marchi S., Montomoli E., Trombetta C. M. (2020). Toxoplasma gondii in women of childbearing age and during pregnancy: seroprevalence study in Central and Southern Italy from 2013 to 2017. *Parasite*.

[5] Feldman D. M., Keller R., Borgida A. F. (2016). Toxoplasmosis, parvovirus, and cytomegalovirus in pregnancy. *Clinics in Laboratory Medicine*.

[6] Storchilo H. R., Rezende H. H. A., Gomes T. C. (2019). Basic heel prick test: inclusion of screening, diagnosis and criteria for early confirmation of congenital infection by Toxoplasma gondii. *Revista do Instituto de Medicina Tropical de São Paulo*.

[7] Anson-Cartwright L., Dawson K., Holmyard D., Fisher S. J., Lazzarini R. A., Cross J. C. (2000). The glial cells missing-1 protein is essential for branching morphogenesis in the chorioallantoic placenta. *Nature Genetics*.

[8] Tscherning-Casper C., Papadogiannakis N., Anvret M. (1999). The trophoblastic epithelial barrier is not infected in full-term placentae of human immunodeficiency virus-seropositive mothers undergoing antiretroviral therapy. *Journal of Virology*.

[9] Guleria I., Sayegh M. (2007). Maternal acceptance of the fetus: true human tolerance. *Journal of immunology*.

[10] Zhang H., Hu X., Liu X., Zhang R., Fu Q., Xu X. (2012). The Treg/Th17 imbalance in Toxoplasma gondii-infected pregnant mice. *American Journal of Reproductive Immunology*.

[11] Arranz-Solís D., Mukhopadhyay D., Saeij J. J. P. (2021). _Toxoplasma_ effectors that affect pregnancy outcome. *Trends in Parasitology*.

[12] Kwak-Kim J., Bao S., Lee S. K., Kim J. W., Gilman-Sachs A. (2014). Immunological modes of pregnancy loss: inflammation, immune effectors, and stress. *American Journal of Reproductive Immunology*.

[13] Ge Y. Y., Zhang L., Zhang G. (2008). In pregnant mice, the infection of Toxoplasma gondii causes the decrease of CD4^+^CD25^+^-regulatory T cells. *Parasite Immunology*.

[14] Chen J., Huang C., Zhu D. (2017). Chinese 1 strain of Toxoplasma gondii excreted-secreted antigens negatively modulate Foxp3 via inhibition of the TGFssRII/Smad2/Smad3/Smad4 pathway. *Journal of Cellular and Molecular Medicine*.

[15] Chen J. L., Ge Y. Y., Zhang J. (2013). The dysfunction of CD4^+^CD25^+^ regulatory T cells contributes to the abortion of mice caused by Toxoplasma gondii excreted-secreted antigens in early pregnancy. *PLoS One*.

[16] Arora N., Sadovsky Y., Dermody T. S., Coyne C. B. (2017). Microbial vertical transmission during human pregnancy. *Cell Host & Microbe*.

[17] Shiono Y., Mun H. S., He N. (2007). Maternal-fetal transmission of _Toxoplasma gondii_ in interferon- *γ* deficient pregnant mice. *Parasitology International*.

[18] Robbins J. R., Zeldovich V. B., Poukchanski A., Boothroyd J. C., Bakardjiev A. I. (2012). Tissue barriers of the human placenta to infection with Toxoplasma gondii. *Infection and Immunity*.

[19] Hunt J. S. (2006). Stranger in a strange land. *Immunological Reviews*.

[20] Vogel P. (2005). The current molecular phylogeny of Eutherian mammals challenges previous interpretations of placental evolution. *Placenta*.

[21] Jena M. K., Nayak N., Chen K., Nayak N. R. (2019). Role of macrophages in pregnancy and related complications. *Archivum Immunologiae et Therapiae Experimentalis (Warsz)*.

[22] Brito C., Silva T., Castro M. (2020). Toxoplasma gondii infection reduces serum progesterone levels and adverse effects at the maternal-foetal interface. *Parasite Immunology*.

[23] Castaño P., Fuertes M., Ferre I. (2014). Placental thrombosis in acute phase abortions during experimental Toxoplasma gondii infection in sheep. *Veterinary Research*.

[24] Fadaam N. (2016). Placental and fetal tissue structural changes resulting from congenital toxoplasmosis. *WORLD JOURNAL OF PHARMACY AND PHARMACEUTICAL SCIENCES*.

[25] Yavuz E., Aydın F., Seyhan A. (2006). Granulomatous villitis formed by inflammatory cells with maternal origin: a rare manifestation type of placental toxoplasmosis. *Placenta*.

[26] Chen J., Liang Y., Yi P. (2017). Outcomes of congenital Zika disease depend on timing of infection and maternal-fetal interferon action. *Cell Reports*.

[27] Liu S., Diao L., Huang C., Li Y., Zeng Y., Kwak-Kim J. Y. H. (2017). The role of decidual immune cells on human pregnancy. *Journal of Reproductive Immunology*.

[28] Nagamatsu T., Schust D. J. (2010). The immunomodulatory roles of macrophages at the maternal-fetal interface. *Reproductive Sciences*.

[29] Xu Y., Romero R., Miller D. (2016). An M1-like macrophage polarization in decidual tissue during spontaneous preterm labor that is attenuated by rosiglitazone treatment. *Journal of Immunology*.

[30] Wakkach A., Fournier N., Brun V., Breittmayer J. P., Cottrez F., Groux H. (2003). Characterization of dendritic cells that induce tolerance and T regulatory 1 cell differentiation in vivo. *Immunity*.

[31] Schumacher A., Wafula P. O., Teles A. (2012). Blockage of heme oxygenase-1 abrogates the protective effect of regulatory T cells on murine pregnancy and promotes the maturation of dendritic cells. *PLoS One*.

[32] Guerrero B., Hassouneh F., Delgado E., Casado J. G., Tarazona R. (2020). Natural killer cells in recurrent miscarriage: an overview. *Journal of Reproductive Immunology*.

[33] Polese B., Gridelet V., Araklioti E., Martens H., Perrier dâ€™Hauterive S., Geenen V. (2014). The endocrine milieu and CD4 T-lymphocyte polarization during pregnancy. *Frontiers in Endocrinology*.

[34] Wilczynski J. R. (2005). Th1/Th2 cytokines balance-- _yin_ and _yang_ of reproductive immunology. *European Journal of Obstetrics, Gynecology, and Reproductive Biology*.

[35] Saito S. (2000). Cytokine network at the feto-maternal interface. *Journal of Reproductive Immunology*.

[36] AbdulHussain G., Azizieh F., Makhseed M.’., Raghupathy R. (2020). Effects of progesterone, dydrogesterone and estrogen on the production of Th1/Th2/Th17 cytokines by lymphocytes from women with recurrent spontaneous miscarriage. *Journal of Reproductive Immunology*.

[37] Chaouat G., Lédée-Bataille N., Zourbas S. (2003). Cytokines, implantation and early abortion: re-examining the Th1/Th2 paradigm leads to question the single pathway, single therapy concept. *American Journal of Reproductive Immunology*.

[38] Zenclussen A. C. (2006). Regulatory T cells in pregnancy. *Springer Seminars in Immunopathology*.

[39] Figueiredo A. S., Schumacher A. (2016). The T helper type 17/regulatory T cell paradigm in pregnancy. *Immunology*.

[40] Liu J. J., Zhang L., Zhang F. F. (2019). Influence of miR-34a on preeclampsia through the Notch signaling pathway. *European Review for Medical and Pharmacological Sciences*.

[41] Ozkan Z. S., Devecı D., Sımsek M., Ilhan F., Rısvanlı A., Sapmaz E. (2015). What is the impact of SOCS3, IL-35 and IL17 in immune pathogenesis of recurrent pregnancy loss?. *The Journal of Maternal-Fetal & Neonatal Medicine*.

[42] Owen M., Clarkson M., Trees A. J. (1998). Acute phase Toxoplasma abortions in sheep. *The Veterinary Record*.

[43] Prigione I., Chiesa S., Taverna P. (2006). T cell mediated immune responses to _Toxoplasma gondii_ in pregnant women with primary toxoplasmosis. *Microbes and Infection*.

[44] Saadatnia G., Mohamed Z., Ghaffarifar F., Osman E., Moghadam Z. K., Noordin R. (2012). Toxoplasma gondii excretory secretory antigenic proteins of diagnostic potential. *APMIS*.

[45] Assossou O., Besson F. Ã.§., Rouault J. P. (2004). Characterization of an excreted/secreted antigen form of 14-3-3 protein in Toxoplasma gondii tachyzoites. *FEMS Microbiology Letters*.

[46] Yang J., Wei P., Barbi J. (2020). The deubiquitinase USP44 promotes Treg function during inflammation by preventing FOXP3 degradation. *EMBO reports*.

[47] Mohr A., Malhotra R., Mayer G., Gorochov G., Miyara M. (2018). Human FOXP3^+^ T regulatory cell heterogeneity. *Clinical & Translational Immunology*.

[48] Dominguez-Villar M., Hafler D. A. (2018). Regulatory T cells in autoimmune disease. *Nature Immunology*.

[49] Georgiev P., Charbonnier L.-M., Chatila T. A. (2019). Regulatory T cells: the many faces of Foxp3. *Journal of Clinical Immunology*.

[50] Kumar V. (2004). Homeostatic control of immunity by TCR peptide-specific Tregs. *The Journal of Clinical Investigation*.

[51] Ke X., Wang J., Li L., Chen I. H., Wang H., Yang X. F. (2008). Roles of CD4+CD25^high^ FOXP3+ Tregs in lymphomas and tumors are complex. *Frontiers in Bioscience*.

[52] Gondek D. C., Lu L. F., Quezada S. A., Sakaguchi S., Noelle R. J. (2005). Cutting edge: contact-mediated suppression by CD4^+^CD25^+^ regulatory cells involves a granzyme B-dependent, perforin-independent mechanism. *Journal of Immunology*.

[53] Zhang B., Chikuma S., Hori S., Fagarasan S., Honjo T. (2016). Nonoverlapping roles of PD-1 and FoxP3 in maintaining immune tolerance in a novel autoimmune pancreatitis mouse model. *Proceedings of the National Academy of Sciences of the United States of America*.

[54] Ghaebi M., Nouri M., Ghasemzadeh A. (2017). Immune regulatory network in successful pregnancy and reproductive failures. *Biomedicine & Pharmacotherapy*.

[55] Pedros C., Zhang Y., Hu J. K. (2016). A TRAF-like motif of the inducible costimulator ICOS controls development of germinal center T_FH_ cells via the kinase TBK1. *Nature Immunology*.

[56] Alijotas-Reig J., Llurba E., Gris J. M. (2014). Potentiating maternal immune tolerance in pregnancy: a new challenging role for regulatory T cells. *Placenta*.

[57] Yang W. Y., Shao Y., Lopez-Pastrana J., Mai J., Wang H., Yang X. F. (2015). Pathological conditions re-shape physiological Tregs into pathological Tregs. *Burns & Trauma*.

[58] Ander S. E., Diamond M. S., Coyne C. B. (2019). Immune responses at the maternal-fetal interface. *Science Immunology*.

[59] Aluvihare V. R., Kallikourdis M., Betz A. G. (2004). Regulatory T cells mediate maternal tolerance to the fetus. *Nature Immunology*.

[60] Wang J., Yang J., Yan Y. (2019). Effect of adoptive transfer of CD4^+^CD25^+^Foxp3^+^ Treg induced by trichostatin A on the prevention of spontaneous abortion. *Journal of Reproductive Immunology*.

[61] Jin L. P., Chen Q. Y., Zhang T., Guo P. F., Li D. J. (2009). The CD4^+^CD25^bright^ regulatory T cells and CTLA-4 expression in peripheral and decidual lymphocytes are down- regulated in human miscarriage. *Clinical Immunology*.

[62] Liu B., Wu H., Huang Q., Li M., Fu X. (2020). Phosphorylated STAT3 inhibited the proliferation and suppression of decidual Treg cells in unexplained recurrent spontaneous abortion. *International Immunopharmacology*.

[63] Pijnenborg R., Bland J. M., Robertson W. B., Brosens I. (1983). Uteroplacental arterial changes related to interstitial trophoblast migration in early human pregnancy. *Placenta*.

[64] Robertson S. A., Care A. S., Moldenhauer L. M. (2018). Regulatory T cells in embryo implantation and the immune response to pregnancy. *The Journal of Clinical Investigation*.

[65] Mellor A. L., Sivakumar J., Chandler P. (2001). Prevention of T cell-driven complement activation and inflammation by tryptophan catabolism during pregnancy. *Nature Immunology*.

[66] Guerin L. R., Prins J. R., Robertson S. A. (2009). Regulatory T-cells and immune tolerance in pregnancy: a new target for infertility treatment?. *Human Reproduction Update*.

[67] Care A. S., Bourque S. L., Morton J. S., Hjartarson E. P., Robertson S. A., Davidge S. T. (2018). Reduction in regulatory T cells in early pregnancy causes uterine artery dysfunction in mice. *Hypertension*.

[68] Ye J., Wang Y., Wang Z. (2018). Circulating Th1, Th2, Th9, Th17, Th22, and Treg levels in aortic dissection patients. *Mediators of Inflammation*.

[69] Nadkarni S., Smith J., Sferruzzi-Perri A. N. (2016). Neutrophils induce proangiogenic T cells with a regulatory phenotype in pregnancy. *Proceedings of the National Academy of Sciences of the United States of America*.

[70] Jasper M. J., Tremellen K. P., Robertson S. A. (2006). Primary unexplained infertility is associated with reduced expression of the T-regulatory cell transcription factor Foxp3 in endometrial tissue. *Molecular Human Reproduction*.

[71] Zhang H., Cui L., Ren L. (2019). The role of decidual PD-1^+^ Treg cells in adverse pregnancy outcomes due to Toxoplasma gondii infection. *Inflammation*.

[72] Oldenhove G., Bouladoux N., Wohlfert E. A. (2009). Decrease of Foxp3^+^ Treg cell number and acquisition of effector cell phenotype during lethal infection. *Immunity*.

[73] Liu Y., Zhao M., Xu X. (2014). Adoptive transfer of Treg cells counters adverse effects of Toxoplasma gondii infection on pregnancy. *The Journal of Infectious Diseases*.

[74] Azizieh F. Y., Raghupathy R. (2017). IL-10 and pregnancy complications. *Clinical and Experimental Obstetrics & Gynecology*.

[75] Zhao M., Zhang R., Xu X. (2013). IL-10 reduces levels of apoptosis in Toxoplasma gondii-infected trophoblasts. *PLoS One*.

[76] Thompson C. D., Zurko J. C., Hanna B. F., Hellenbrand D. J., Hanna A. (2013). The therapeutic role of interleukin-10 after spinal cord injury. *Journal of Neurotrauma*.

[77] Lao K., Zhao M., Li Z. (2015). IL-10 regulate decidual Tregs apoptosis contributing to the abnormal pregnancy with _Toxoplasma gondii_ infection. *Microbial Pathogenesis*.

[78] Qiu J., Zhang R., Xie Y. (2018). Estradiol attenuates the severity of primary toxoplasma gondii infection-induced adverse pregnancy outcomes through the regulation of Tregs in a dose-dependent manner. *Frontiers in Immunology*.

[79] Olguín J. E., Fernández J., Salinas N. (2015). Adoptive transfer of CD4^+^Foxp3^+^ regulatory T cells to C57BL/6J mice during acute infection with _Toxoplasma gondii_ down modulates the exacerbated T_h_1 immune response. *Microbes and Infection*.

[80] Mitsuiki N., Schwab C., Grimbacher B. (2019). What did we learn from CTLA-4 insufficiency on the human immune system?. *Immunological Reviews*.

[81] Francisco L. M., Sage P. T., Sharpe A. H. (2010). The PD-1 pathway in tolerance and autoimmunity. *Immunological Reviews*.

[82] Jonuleit H., Schmitt E., Kakirman H., Stassen M., Knop J.¨., Enk A. H. (2002). Infectious tolerance. *The Journal of Experimental Medicine*.

[83] Wang S., Sun F., Li M. (2019). The appropriate frequency and function of decidual Tim-3^+^CTLA-4^+^CD8^+^ T cells are important in maintaining normal pregnancy. *Cell Death & Disease*.

[84] Miwa N., Hayakawa S., Miyazaki S. (2005). IDO expression on decidual and peripheral blood dendritic cells and monocytes/macrophages after treatment with CTLA-4 or interferon-*γ* increase in normal pregnancy but decrease in spontaneous abortion. *Molecular Human Reproduction*.

[85] Wafula P. O., Teles A., Schumacher A. (2009). Original article: PD-1 but not CTLA-4 blockage abrogates the protective effect of regulatory T cells in a pregnancy murine model. *American Journal of Reproductive Immunology*.

[86] Habicht A., Dada S., Jurewicz M. (2007). A link between PDL1 and T regulatory cells in fetomaternal tolerance. *Journal of Immunology*.

[87] D’Addio F., Riella L. V., Mfarrej B. G. (2011). The link between the PDL1 costimulatory pathway and Th17 in fetomaternal tolerance. *Journal of Immunology*.

[88] Si L. F., Zhang S. Y., Gao C. S., Chen S. L., Zhao J., Cheng X. C. (2013). Effects of IFN-*γ* on IL-18 expression in pregnant rats and pregnancy outcomes. *Asian-Australasian Journal of Animal Sciences*.

[89] Lu L., Barbi J., Pan F. (2017). The regulation of immune tolerance by FOXP3. *Nature Reviews. Immunology*.

[90] Hou W., Li Z., Li Y. (2016). Correlation between protein expression of FOXP3 and level of FOXP3 promoter methylation in recurrent spontaneous abortion. *The Journal of Obstetrics and Gynaecology Research*.

[91] Liu M., Li S., Li M. O. (2018). TGF-*β* control of adaptive immune tolerance: a break from Treg cells. *Bioessays*.

[92] Tang Y.-J., Xiao J., Huang X. R. (2014). Latent transforming growth factor-*β*1 protects against bleomycin-induced lung injury in mice. *American journal of respiratory cell and molecular biology*.

[93] Pang N., Zhang F., Ma X. (2014). TGF-*β*/Smad signaling pathway regulates Th17/Treg balance during _Echinococcus multilocularis_ infection. *International Immunopharmacology*.

[94] Zhao M., Zhang H., Liu X., Jiang Y., Ren L., Hu X. (2017). The effect of TGF-*β* on Treg cells in adverse pregnancy outcome upon Toxoplasma gondii infection. *Frontiers in Microbiology*.

[95] Chinen T., Kannan A. K., Levine A. G. (2016). An essential role for the IL-2 receptor in T_reg_ cell function. *Nature Immunology*.

[96] Goldstein J. D., Burlion A., Zaragoza B. (2016). Inhibition of the JAK/STAT signaling pathway in regulatory T cells reveals a very dynamic regulation of Foxp3 expression. *PLoS One*.

[97] Chen J., Huang C., Zhu D. (2018). Excreted-secreted antigens of Toxoplasma gondii inhibit Foxp3 via IL-2R*γ*/JAK3/Stats pathway. *Journal of Cellular Biochemistry*.

[98] Gao L., Dong Y., Lin R., Meng Y., Wu F., Jia L. (2020). The imbalance of Treg/Th17 cells induced by perinatal bisphenol A exposure is associated with activation of the PI3K/Akt/mTOR signaling pathway in male offspring mice. *Food and Chemical Toxicology*.

[99] O’Brien C. A., Harris T. H. (2020). ICOS-deficient and ICOS YF mutant mice fail to control Toxoplasma gondii infection of the brain. *PLoS One*.

[100] Ouyang W., Beckett O., Ma Q., Paik J. H., DePinho R. A., Li M. O. (2010). Foxo proteins cooperatively control the differentiation of Foxp3^+^ regulatory T cells. *Nature Immunology*.

[101] Chen J., Hu L., Wang J. (2019). Toxoplasma gondii excreted-secreted antigens suppress Foxp3 via PI3K-AKT-mTOR signaling pathway. *Journal of Cellular Biochemistry*.

[102] Flegr J. (2013). How and why _Toxoplasma_ makes us crazy. *Trends in Parasitology*.

[103] Lim A., Kumar V., Hari Dass S. A., Vyas A. (2013). Toxoplasma gondii infection enhances testicular steroidogenesis in rats. *Molecular Ecology*.

[104] Xiao J., Li Y., Prandovszky E. (2014). MicroRNA-132 dysregulation in _Toxoplasma gondii_ infection has implications for dopamine signaling pathway. *Neuroscience*.

[105] Miller B. J., Goldsmith D. R. (2017). Towards an immunophenotype of schizophrenia: progress, potential mechanisms, and future directions. *Neuropsychopharmacology: official publication of the American College of Neuropsychopharmacology*.

[106] Hellmer K., Nystrom P. (2017). Infant acetylcholine, dopamine, and melatonin dysregulation: neonatal biomarkers and causal factors for ASD and ADHD phenotypes. *Medical Hypotheses*.

[107] Najjar S., Pearlman D. M. (2015). Neuroinflammation and white matter pathology in schizophrenia: systematic review. *Schizophrenia Research*.

[108] Contreras F., Prado C., González H. (2016). Dopamine receptor D3 signaling on CD4^+^T cells favors Th1- and Th17-mediated immunity. *Journal of Immunology*.

[109] Cosentino M., Ferrari M., Kustrimovic N., Rasini E., Marino F. (2015). Influence of dopamine receptor gene polymorphisms on circulating T lymphocytes: a pilot study in healthy subjects. *Human Immunology*.

[110] Osorio-Barrios F., Prado C., Contreras F., Pacheco R. (2018). Dopamine receptor D5 signaling plays a dual role in experimental autoimmune encephalomyelitis potentiating Th17-mediated immunity and favoring suppressive activity of regulatory T-cells. *Frontiers in Cellular Neuroscience*.

[111] Anderson G., Berk M., Dodd S. (2013). Immuno-inflammatory, oxidative and nitrosative stress, and neuroprogressive pathways in the etiology, course and treatment of schizophrenia. *Progress in Neuro-Psychopharmacology and Biological Psychiatry*.

[112] Xu Z., Zhang X., Chang H. (2021). Rescue of maternal immune activation-induced behavioral abnormalities in adult mouse offspring by pathogen-activated maternal T_reg_ cells. *Nature Neuroscience*.

[113] Clark D. A. (2016). The importance of being a regulatory T cell in pregnancy. *Journal of Reproductive Immunology*.

